# Collagen Analogs Promote Tissue Regeneration in HSV-1-Infected Corneas in Animal Models

**DOI:** 10.3390/jfb16100377

**Published:** 2025-10-09

**Authors:** Oleksiy Buznyk, Hamid Goodarzi, Jaime Gómez Laguna, Jaganmohan Reddy, Aneta Liszka, Elle Edin, Christos Boutopoulos, James Chodosh, Mohammad Mirazul Islam, May Griffith

**Affiliations:** 1 Department of Biomedical and Clinical Sciences, Linköping University, SE-581 83 Linköping, Sweden; oleksiy.buznyk@regionostergotland.se (O.B.); jjmreddy@gmail.com (J.R.); aneta.liszka@liu.se (A.L.); elle.edin@biomimir.ca (E.E.); 2 Department of Ophthalmology, Linköping University, SE-581 85 Linköping, Sweden; 3 Filatov Institute of Eye Diseases & Tissue Therapy of the NAMS of Ukraine, 65015 Odessa, Ukraine; 4 Maisonneuve-Rosemont Hospital Research Centre (CRHMR), Montreal, QC H1T 2H2, Canada; hamid.goodarzi@umontreal.ca (H.G.); christos.boutopoulos@umontreal.ca (C.B.); 5 Department of Ophthalmology and Institute of Biomedical Engineering, Université de Montréal, Montreal, QC H3T 1J4, Canada; 6 Department of Anatomy and Comparative Pathology and Toxicology, Pathology and Immunology Group (UCO-PIG), UIC Zoonosis y Enfermedades Emergentes ENZOEM, International Excellence Agrifood Campus ‘CeiA3’, University of Córdoba, 14014 Córdoba, Spain; v92golaj@uco.es; 7 UR Advanced Therapeutics, Hyderabad 501203, Telangana, India; 8 Department of Ophthalmology and Visual Sciences, University of New Mexico, Albuquerque, NM 87106, USA; jchodosh@salud.unm.edu

**Keywords:** HSV-1, cornea, biosynthetic corneal implant, rabbit, guinea pig

## Abstract

Herpes simplex virus type 1 (HSV-1) is a leading cause of infectious corneal blindness worldwide. Human donor corneal transplantation remains the primary treatment for scarred corneas resulting from herpes simplex keratitis (HSK), a severe inflammatory corneal disease caused by HSV-1 infection, despite a high risk of re-infection or immune rejection of the allografts. As possible alternatives to donor grafting for HSK, we developed cell-free, regeneration-stimulating corneal implants designed to work even under adverse inflammatory situations such as severe infections. The implants comprised short, fully synthetic collagen-like peptides conjugated to polyethylene glycol (CLP-PEG) and crosslinked using carbodiimide chemistry. Being cell-free, they lacked the cellular targets that an already activated immune system would encounter in these inflamed corneas. We tested the performance of these implants in guinea pig and rabbit models of HSK. Three different HSV-1 strains were used to create experimental HSK in rabbits and guinea pigs. There were no overall statistically significant species differences or species–strain differences in virus-induced mortality. At three months post-operation, all treated corneas showed tissue regeneration, but with haze or neovascularization. The initially cell-free CLP-PEG implants allowed for repopulation by ingrowing cells to regenerate neocorneal tissue, despite the inflammation. However, they did not prevent HSV-1 reactivation nor re-infection, as neovascularization and disorganization were observed within the neocorneas. A detailed histopathological examination revealed viral strain differences, but only KOS infection showed interspecies neovascularization differences. A more detailed examination with larger numbers of animals is merited to fully elucidate the effects of the different viral strains on rabbits versus guinea pigs.

## 1. Introduction

Engineered biomaterials for healthcare to enhance or repair damaged organs and promote regeneration are entering the clinic at unprecedented rates [1]. However, biomaterials grafted into patients are often inadequate in resisting infections and break down due to the body’s inflammatory responses [2]. The recent severe acute respiratory syndrome coronavirus 2 (SARS-CoV-2) pandemic produced inflammation that was pervasive throughout the body, clearly showing an urgent need for new biomaterials that promote regeneration in organs despite ongoing active infections. Our goal, therefore, is to develop robust biomaterials capable of promoting organ regeneration despite active infections and inflammation, using the cornea as our model.

The cornea is the clear outer window of the eye that transmits light into the interior for vision [3]. It comprises three main cellular layers: an outermost epithelium comprising five to six stratified layers and a single innermost endothelial layer sandwiching a stroma of mainly collagenous extracellular matrix with interconnected cells arranged in layers or lamellae [3]. The cornea is avascular, allowing for transparency, and receives significant trophic support from its dense innervation [4]. Infections or injuries causing permanent loss of transparency can result in blindness [5]. The only widely accepted treatment for corneal blindness is donor allograft transplantation. Unfortunately, over 50% of countries have no transplantation capacity, and among the rest, a severe shortfall leaves only 1 out of 70 or 1.4% of patients receiving a donor cornea [6]. However, even if donor tissues are readily available, up to 50% of grafts will be rejected if the recipient cornea is inflamed [7].

Corneal inflammation often results from infection, and herpes simplex virus type 1 (HSV-1) is a major cause of corneal infection and inflammation. Its seroprevalence is as high as 75% in developed countries [8,9]. The human cornea being particularly susceptible to this infection, making it a leading infectious cause of blindness [10]. Human donor corneal transplantation remains the primary treatment for severe corneal disease caused by herpes simplex keratitis (HSK) due to HSV-1 infection, despite a high risk of viral reactivation and immune rejection, reportedly as high as 59% in one study [11,12].

We previously developed corneal implants of short collagen-like peptides (CLPs) conjugated to polyethylene glycol (PEG) as analogs of collagen, the main structural biomaterial of the cornea [13]. The CLP-PEG implants were designed cell-free so there would not be any cellular targets for the activated immune systems in inflamed corneas. For HSV-1, reactivation of the latent virus during surgery has been reported, with an overall rate of recurrent dendritic keratitis after transplantation for herpes at 19% in a retrospective study of 107 penetrating keratoplasties [11]. Any proposed clinically relevant implant would need to promote regeneration despite an active infection. While CLP-PEG implants are as effective at promoting regeneration [13] as their full-length collagen counterparts [14], their performance in corneas with active infections has not been evaluated.

Here, we evaluated the ability of CLP-PEG collagen analog implants to promote regeneration in rabbit and guinea pig corneas affected by HSK. Three different HSV-1 strains were tested to determine if the different viruses had any differential effects on the regenerative outcomes of treatments in the two different animal models. This will provide guidance for the selection of animal models and viral strains for future studies.

## 2. Materials and Methods

### 2.1. CLP-PEG Implants Fabrication

Briefly, CLP peptide [CG(PKG)4(POG)4(DOG)4] (Genscript, Piscataway, NJ, USA and UAB Ferentis, Vilnius, Lithuania) was conjugated to 8-arm poly(ethylene glycol) maleimide (PEG-maleimide, Sinopeg Biotech Co. Ltd., Xiamen, China) at pH 4.5 by mixing them at a ratio of 5:1. The resulting CLP-PEG solution was sterile-filtered and dialyzed using a 10–12 kDa cut-off dialysis membrane and then lyophilized and redissolved in 2-(N-morpholino)ethanesulfonic acid (MES) buffer (0.625 M, pH 4.5). CLP-PEG (12% *w*/*w*) was then crosslinked with 1-ethyl-3-[3-dimethylaminopropyl] carbodiimide hydrochloride (EDC) and N-hydroxysuccinimide (NHS) (Sigma-Aldrich, St. Louis, MO, USA). The molar equivalent ratio of EDC to CLP-PEG amine was 1:2, and the molar ratio of EDC:NHS was 1:1. After thorough mixing, liquid hydrogel was dispensed into 10 mm diameter, cornea-shaped molds (Formteknik, Anderstorp, Sweden) secured in stainless steel jigs and cured for 48 h at room temperature in a humidified chamber. Subsequently, the implants were demolded and stored in 0.1 M sterile phosphate-buffered saline (PBS) containing 1% chloroform at 4–8 °C to maintain sterility [15,16,17]. Before surgery, the implants were washed in PBS containing 0.5% gentamicin to remove the chloroform and eliminate any residual contamination, after which antibiotics were washed away. The resulting implants were characterized for their chemical, physical, and mechanical properties, as previously reported [14].

### 2.2. Characterization

#### 2.2.1. FTIR-ATR and NMR

For chemical characterization, samples were first dried for 24 h in a vacuum desiccator to remove moisture and volatile impurities. FTIR analysis was performed using a Fourier-Transform Infrared Spectrometer (FTIR, Nicolet 6700/Smart iTR model, Thermo Scientific, Waltham, MA, USA) with a ZnSe or diamond ATR crystal. The dried samples were placed directly on the crystal without dilution in a KBr matrix. For each sample, 32 scans were collected at a resolution of 4 cm^−1^ over a range of 4000–400 cm^−1^.

Solid-state NMR analysis was conducted using a 400 MHz spectrometer (Bruker Avance III HD, Billerica, MA, USA). Approximately 40 mg of each dried sample was loaded into the sampling tube. For each sample, 2048 scans were acquired over a chemical shift range of 0–200 ppm.

#### 2.2.2. Scanning Electron Microscopy

The implants were equilibrated for 24 h in water to achieve full hydration, after which they were freeze-dried. The samples were then fractured under liquid nitrogen to create cross-sections, followed by chromium sputter-coating (using the Q150R-ES, Quorum Technologies, England) for enhanced imaging. The implant’s structure was analyzed using field emission gun scanning electron microscopy (FEG-SEM, JSM-7600F model, JEOL Ltd., Tokyo, Japan).

#### 2.2.3. Water Content and Collagenase Degradation

To determine the water content, implants (*n* = 3) were blotted to remove excess water and then weighed to obtain the starting “wet weight” (W_0_) of the implant. The samples were then dried under a vacuum until a stable “dry weight” (W_d_) was obtained. The total equilibrated water content of the hydrogels (W) was calculated according to the equation

W% = (W_0_ − W_d_)/W_0_ × 100%

To determine susceptibility to collagenase degradation, each implant was equilibrated in Tris-HCl buffer (0.1 M, pH 7.4) overnight, blotted dry, and weighed (W_0_). The implants (*n* = 3) were then incubated in 55 U/mL Type I bacterial collagenase dissolved in Tris-HCl at 37 °C. The collagenase solution was replaced with a fresh solution every 8 h. The undigested weight (W_t_) was determined at 14 days. The percentage of mass remaining after digestion was calculated according to the following equation: residual mass (%) = (W_t_/W_0_) × 100%.

#### 2.2.4. Optical Properties

The refractive index of the hydrogel was measured at RT on a refractometer (Abbemat 300, Anton Parr, model 5, Bellingham & Stanley Ltd., Tunbridge Wells, UK). Light transmission and backscatter through the hydrogels were measured using a custom-designed device employing a stable tungsten–halogen light source (SLS201L, Thorlabs, Newton, NJ, USA) in conjunction with an infrared filter (FESH0750, Thorlabs). Measurements were conducted at RT using flat sheets (9 mm in diameter and 0.5 mm in thickness) made of the hydrogel. The light (360 nm–750 nm) was coupled into a fiber bundle (RP26, Thorlabs), and a lens (ACL25416U-A, Thorlabs) was used to generate a collimated beam with a diameter of 9 mm. Samples were placed in the beam path inside an optical isolating enclosure. A power sensor (PM16-120, Thorlabs) was utilized to measure the optical transmission of the samples, and a second power sensor (PM16-130, Thorlabs) was used to offset any drift in the output power of the light source.

### 2.3. Differential Scanning Calorimeter (DSC)

Differential scanning calorimetry (DSC) was performed using a DSC 25 (TA Instruments, New Castle, DE, USA) instrument. Each sample (2–5 mg) was put inside sealed aluminum pans (T zero) and run at a scanning rate of 10 °C/min.

### 2.4. Physical Properties of Implants

Rheology and tensile strength of the implants were measured as follows.

A Discovery Hybrid-2 rheometer (TA Instruments, New Castle, DE, USA) fitted with an 8 mm circular geometry was used to obtain the storage modulus (G’) of the hydrogel. An amplitude sweep was run from 0.1 to 200% using an angular frequency of 10 rad/s. Molded dogbone-shaped hydrogels (length: 13 mm, width: 7 mm, thickness: 0.5 mm) were evaluated for mechanical strength using an Instron electromechanical universal tester (Model 3342, Instron, Norwood, MA, USA) equipped with the Series IX/S software. Young’s modulus and tensile strength were tested at 20 °C and 50% humidity using a crosshead speed of 10 mm/min.

### 2.5. HSV-1 Strains

HSV-1 strains F and KOS 321P5 were purchased from ATCC (Manassas, VA, USA), and a clinical strain isolated from a patient with HSV keratitis (i38) was kindly provided by Dr. Bruce Jackson (University of Ottawa Eye Institute, Ottawa, ON, Canada) (Table 1). The HSV-1 strains were propagated and titered according to ATCC directions, while i38 was propagated in Vero cells using standardized protocols [18,19]. Table 1 shows the different stains tested.

### 2.6. Animals, HSV-1 Corneal Infections, and Surgical Implantation

There are no sex differences for HSV-1 infections noted for animals or humans, so males, which are more docile for handling, were used [10,20]. Ethical permission for the study was obtained from the north Stockholm animal research ethics committee, “Stockholms norra djurförsöksetiska nämnd” (DNR N30/14).

The HSV-1 strains F, KOS 321P5, and i38 were used to create experimental corneal HSK in 12 male rabbits and 12 male guinea pigs (4 animals per virus strain). Corneas were marked centrally with a trephine (5 mm for rabbits, 3 mm for guinea pigs) in each animal’s right cornea, and the epithelium was removed within the mark using a diamond knife. An inoculum of 2 × 10^5^ PFU of HSV in 10 µL of sterile 0.01 M PBS was introduced into each wound. This selected dose was previously reported to promote inflammation and induce latency in rabbit corneas [21]. After 30 s, the corneal surface was washed with saline and allowed to heal for seven weeks to run the course of the infection.

After the pre-operation evaluation, the infected corneas of each surviving animal underwent anterior lamellar keratoplasty (ALK) implantation, leaving a thin layer of deep stroma, with CLP-PEG implants, all performed by a single surgeon (OB). For rabbits, the implants were 6 mm in diameter and 350 µm thick; for guinea pigs, the implants were 3 mm in diameter and 150 µm in thickness. ALK involved the use of a guarded trephine to make a circular cut down to a 70–80% depth. Tissue was then removed by manual lamellar dissection using a crescent knife. After excision of the corneal button, implants were placed in the resulting wound bed and secured with 2–3 overlying 10–0 nylon sutures. Immediately after the surgery, hydrocortisone 10 mg/g–oxytetracycline 5 mg/g ointment (Pfizer AB, Sollentuna, Sweden) was instilled into the implanted eye. Post-operatively, the eyes were treated with tobramycin 3 mg/mL and dexamethasone 1 mg/mL topically twice daily for one week.

### 2.7. Clinical Evaluations

Animals were clinically evaluated before HSV-1 inoculation, at 7 weeks after virus inoculation, just before the surgery, and 3 months after surgery. Slit-lamp biomicroscopy was used to evaluate the corneas and the implants for optical clarity, corneal neovascularization, conjunctival injection, and aqueous humor cell and flare compared to the unoperated and uninfected contralateral control eyes using a modified MacDonald–Shadduck scoring system [22]. Corneal fluorescein staining was used to assess the integrity of the corneal epithelial surface.

Seven weeks after the virus inoculation and right before surgery, the infected corneas were clinically assessed again, as described above.

Animals were followed up for 3 months after implantation, and sutures were removed during the last follow-up for final examinations. The final examinations performed are the same ones performed pre-surgically, plus intraocular pressure (IOP; TonoVet, Icare Finland Oy, Vantaa, Finland), ultrasound pachymetry (Tomey SP 3000, Tomey, Inc., Nagoya, Japan), and in vivo confocal microscopy (IVCM; HRT3 with Rostock Cornea Module, Heidelberg Engineering GmbH, Dossenheim, Germany).

### 2.8. Histopathology and Immunohistochemistry

After the final clinical examination, the animals were euthanized. Both treated and untreated control contralateral corneas with 2 mm of sclera rim were excised and fixed in 4% paraformaldehyde in 0.01 M PBS, pH 7.2–7.4. Each cornea was then divided in half, with one half embedded in paraffin and sectioned for hematoxylin and eosin staining (H&E) and the other half frozen for immunohistochemistry. Then, 7 µm frozen sections of infected and control contralateral corneas were mounted on glass slides (Superfrost Plus Microscope Slides, Thermo Scientific, Waltham, MA, USA), air-dried, and fixed in 4% paraformaldehyde (10 min at 4 °C) and ice-cold methanol (20 min at −20 °C). They were then washed in 0.01 M PBS, permeabilized with 0.3% Triton X100 in 0.01 M PBS, and blocked with 5% goat serum in 0.01 M PBS with 0.1% saponin (Sigma Aldrich, St. Louis, MO, USA) (blocking solution) for 60 min at RT. Incubation with anti-HSV antibody (ab860, Abcam, Cambridge, UK) diluted to 1:100 with the blocking solution was carried out overnight at 4 °C. All slides were washed in 0.01 M PBS with 5% fetal bovine serum (FBS) and 0.1% saponin (washing solution) and then incubated with secondary antibody (Alexa Fluor^®^ 488 goat anti-mouse IgG, Life Technologies, Carlsbad, CA, USA) diluted to 1:1000 with the blocking solution for 60 min at RT. After washing in washing solution, the slides were dehydrated and mounted with Vectashield mounting medium with DAPI (Vector Laboratories, Inc., Burlingame, CA, USA) and imaged with an LSM-800 Zeiss confocal microscope (LSM800, Carl Zeiss, Oberkochen, Germany).

Both right (OD) and left (OS) corneas, infected and control (non-infected), respectively, were blindly evaluated by a certified veterinary pathologist (JGL) for the presence of stromal inflammatory infiltrates, neovascularization, stromal disorganization, lipid deposits, and epithelial hyperplasia. Inflammatory infiltrate, stromal disarrangement, and epithelial hyperplasia were scored as follows: 0, absent; 1, mild; 2, moderate; and 3, severe. Other findings, such as the presence of hemorrhage or retrocorneal fibrovascular proliferation, were also noted.

### 2.9. Statistical Analysis

Where applicable, data was expressed as mean ± standard deviation. Differences in animal survival by species were tested using Fisher’s exact test. Survival rates by strain are reported descriptively due to the small numbers of animals infected per strain per species (*n* = 4). Because early mortality reduced per-group sample sizes, clinical scores (haze, neovascularization) are also reported descriptively. For pachymetry, each eye was measured in multi-point mode, yielding 8 readings from distinct corneal locations per scan. These were averaged to obtain a single value per eye. Pachymetry was measured only post-operatively in the treated eyes. Therefore, no statistical comparison between groups was performed. Values are reported descriptively.

## 3. Results

### 3.1. CLP-PEG Hydrogels

**Figure 1:**
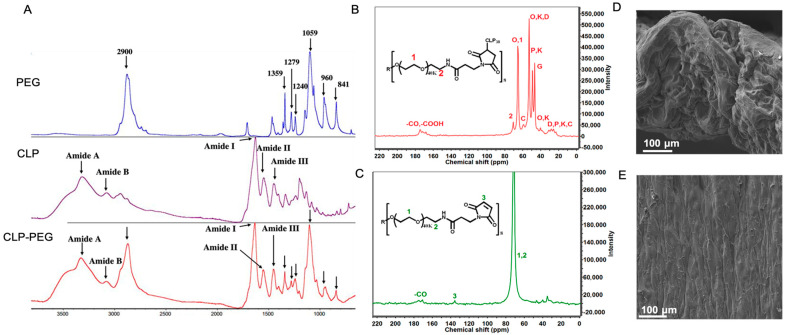
(**A**) FTIR spectra of CLP, PEG, and CLP-PEG conjugate. The spectra display characteristic peaks indicating the successful conjugation of PEG to CLP. (**B**,**C**) Carbon nuclear magnetic resonance (C-NMR) spectra of CLP-PEG conjugate, showing the chemical shifts and corresponding structural assignments. Key peaks are labeled to highlight the successful conjugation process. (**D**,**E**) Scanning electron microscope images of the cross-sectional and surface morphology of the CLP-PEG implant. The cross-sectional view (**D**) shows the internal structure, while the surface view (**E**) reveals the outer morphology, both indicating a relatively uniform and well-formed material. Scale, 100 μm.

Fourier-Transform Infrared Spectrometry (FTIR) and nuclear magnetic resonance (NMR) confirmed the successful CLP-PEG conjugation (Figure 1A–C). Surface observations using scanning electron microscopy confirmed a highly uniform surface, while a cross-section through the hydrogel showed a dense porous structure, that is, structural elements that show the hydrogel is conducive to cell attachment and proliferation (Figure 1D,E).

Table 2 shows the physical and mechanical properties of the CLP-PEG hydrogels. The water content of the hydrogels was 87.5 ± 3.87%. The transmission of white light (400–700 nm) through them averaged 91.44 ± 4.78%, with backscattering at 0.65 ± 0.04%, indicating a very transparent hydrogel. The implant showed a very gradual degradation of 15.82% loss of mass after two weeks of exposure to high concentrations of collagenase (Table 2). This indicates that the implant maintains its structural integrity and resists enzymatic breakdown over an extended period, confirming its suitability for long-term applications in adverse environments, for example, during HSV-1 infection [23].

Rheology findings indicate that the CLP-PEG hydrogels exhibited a linear viscoelastic region (LVR) of 53% ± 7.81%. This indicates that the material deformed by 53% of its initial size in response to the rotational forces applied. A larger LVER suggests that the material can absorb significant deformations before behaving nonlinearly. Additionally, the measured storage modulus was 31.13 ± 2.41 kPa. This value indicates the stiffness of the material and its ability to store mechanical energy when subjected to deformation (Table 2). The tensile strength was 24.12 ± 3.38 kPa, with Young’s modulus at 41.14 ± 6.54 kPa and elongation at break at 42.97 ± 2.12%, showing a robust and flexible hydrogel that was implantable.

### 3.2. HSV-1 Infection in Rabbit and Guinea Pig Corneas

**Figure 2:**
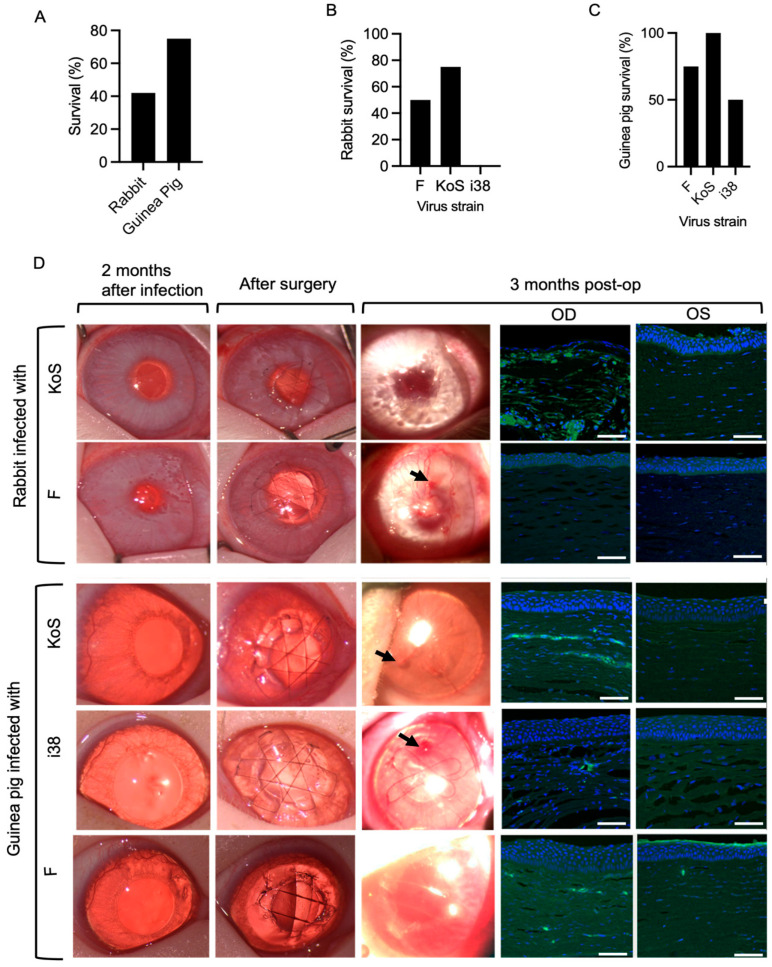
(**A**) Survival outcomes between rabbits and guinea pigs following HSV-1 infection (*p* > 0.05, two-sided Fisher’s exact test). Species–viral strain interaction analysis of survival outcomes following HSV-1 infection in rabbits (**B**) and guinea pigs (**C**). (**D**) Clinical photographs of operated rabbit and guinea pig corneas at 2 months post-infection, immediately after surgery, and at 3 months post-operatively. Arrows indicate epithelial irregularities in the regenerated corneal surface at 3 months post-operation. Sections through the regenerated right (OD) corneas of both rabbits and guinea pigs at 3 months post-operation show the presence of HSV-1 as green staining detected by an antibody against the virus. The non-infected, non-operated left (OS) corneas were unstained. Scale bars, 100 µm.

HSV-1 eye infections were accompanied by encephalitis symptoms such as loss of coordination and seizures in several animals during the first two weeks of post-infection, and these animals were euthanized. Of the 24 animals, 5 out of 12 rabbits infected with F- and KOS-strain HSV-1 survived, while 9 out of 12 guinea pigs infected with all three strains survived (Figure 2A). However, a Fisher’s exact test used to compare survival rates between rabbits and guinea pigs showed that there was no overall significant difference between the survival rates of the two species (*p* = 0.2138). Within the rabbit groups, survival rates were 50% for strain F, 75% for KOS, and 0% for i38 (Figure 2B). In guinea pigs, survival rates were 75% for strain F, 100% for KOS, and 50% for i38 (Figure 2C).

HSK occurred in all the inoculated eyes of the rabbits and guinea pigs, presenting as a mild corneal haze (grade 1–2) and neovascularization within 7–14 days after inoculation. The HSK indications persisted through the two-month post-infection recovery period (Figure 2D).

### 3.3. Clinical Evaluation of Implants in HSV-1-Infected Rabbit and Guinea Pig Corneas

Implantation of CLP-PEG corneal substitutes was well tolerated in all 14 grafted animals over the three-month post-operation follow-up. One i38-infected guinea pig cornea perforated during trephination (surgical mishap) but was repaired by suturing with two single 10/0 nylon sutures before the implant was secured in place.

Peri-surgical inflammation resolved within one to two weeks, and all implants were stably integrated over the three-month follow-up period. Gross examination of the corneas at three months post-operation showed the presence of haze and neovascularization of various degrees (Figure 2D). All the grafted cell-free implants were re-epithelialized (Figure 2D). However, irregularities were observed in the regenerated corneal epithelial surfaces in clinical photographs of the operated corneas at three months post-operation (Figure 2D). Immunohistochemical staining for HSV-1 in sections through the regenerated neocorneas showed the presence of the virus, particularly in the stromal compartments. The most prominent HSV-1-positive staining was found in KOS-infected corneas (Figure 2D).

**Figure 3:**
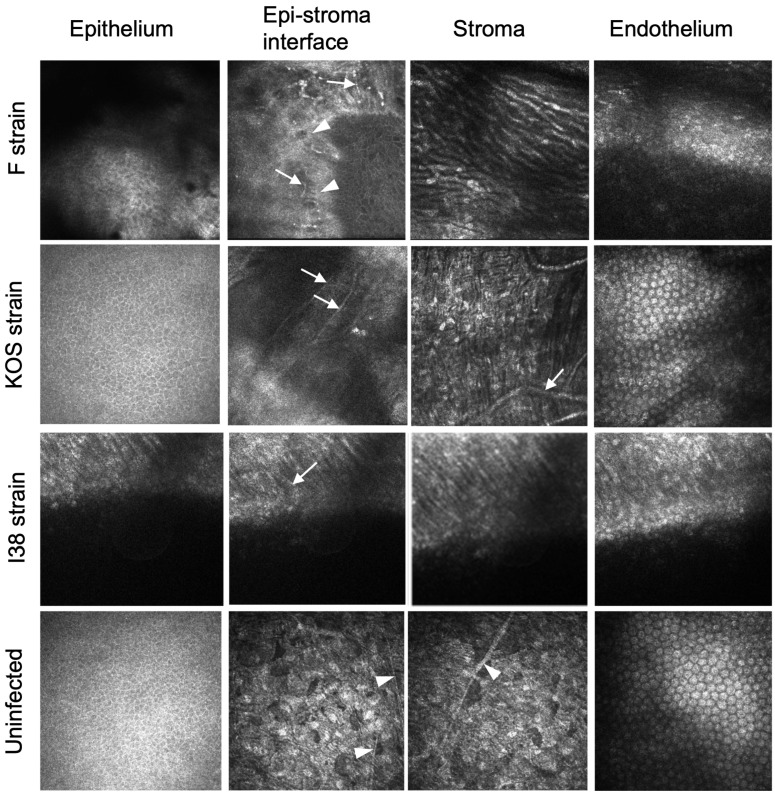
In vivo confocal microscopical images three months after grafting of CLP-PEG implants into guinea pig eyes infected with HSV-1. A control healthy, uninfected cornea is shown for comparison. White arrows indicate blood vessels, while white arrowheads indicate nerves.

IVCM of rabbits was not performed. IVCM of guinea pig corneas showed qualitative differences between the implanted corneas in eyes infected by the different viral strains (Figure 3). Regenerated neocorneas had epitheliums. F-strain-infected eyes exhibited superficial neovascularization, and the stroma contained elongated cells. KOS-strain-infected eyes exhibited normal epithelial and endothelial layers, with very fine superficial neovascularization, but with stromal neovascularization. Implanted corneas of i38-strain-infected eyes showed an irregular epithelium, with fine superficial neovessels. The stroma appeared disorganized but with early cellular repopulation. The sub-epithelial nerve plexus seen at the epithelial–stroma interface in uninfected and unoperated corneas was absent in most implanted eyes. However, several subepithelial nerves were seen in an F-strain-infected, implanted cornea, but only where there was a fine network of blood vessels.

The clinical examination findings for the HSV-1-infected animal corneas before and after CLP-PEG hydrogel implantation are summarized in Table 3. Haze scores did not show clear pre-/post-operative changes within rabbits or within guinea pigs, and interspecies differences in haze were not apparent. Post-operatively, neovascularization scores appeared higher in guinea pig corneas than in rabbit corneas (Table 3). Pachymetry and intraocular pressure in the right eyes were similar to those of the contralateral untreated and uninfected left eyes at three months post-operation.

### 3.4. Histopathological and Immunohistochemical Results

**Figure 4:**
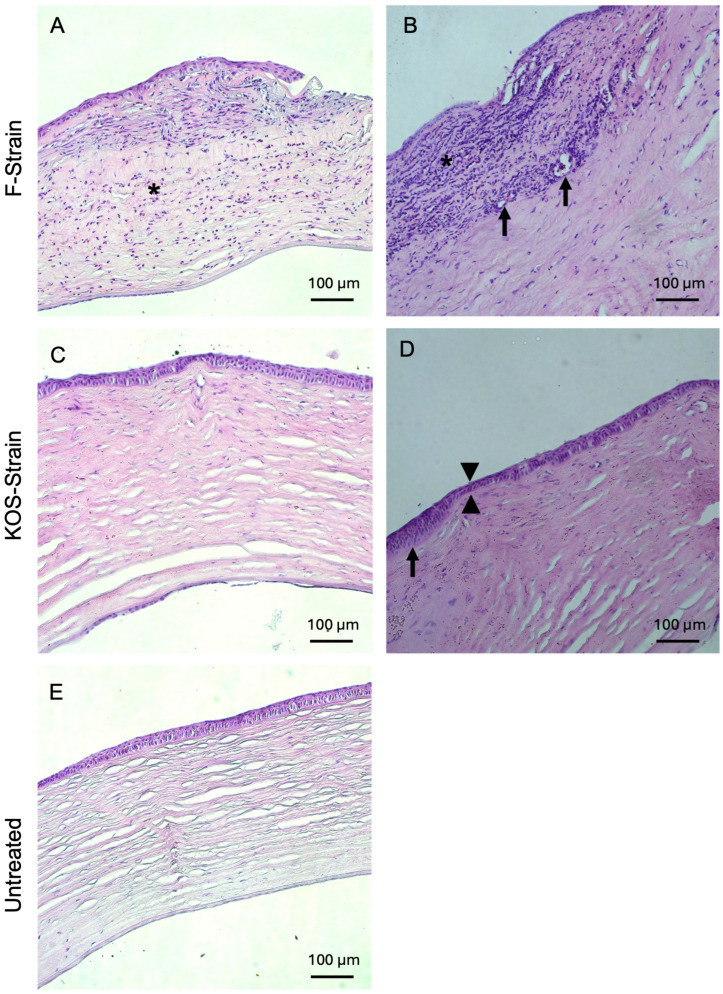
Hematoxylin and eosin-stained corneas from HSV-1-infected rabbits after CLP-PEG implantation at 3 months post-operation, compared with untreated control corneas. Representative sections from F-strain-infected corneas show moderate, diffuse, mixed inflammatory infiltration (asterisk) with epithelial thinning and focal ulceration of the corneal epithelium (**A**) and severe, diffuse, mixed inflammatory infiltrate (asterisk). There was also neovascularization (arrows) and mild stromal disarrangement, as well as epithelial thinning and ulceration of the epithelium (**B**). Corneas infected with the KOS strain only presented mild stromal disorganization and focal epithelial thinning (arrowhead) (**C**), as well as moderate epithelial hyperplasia (arrow) together with epithelial thinning (arrowheads) (**D**). Representative section of an untreated control cornea (**E**). Scale bars, 100 µm.

**Figure 5:**
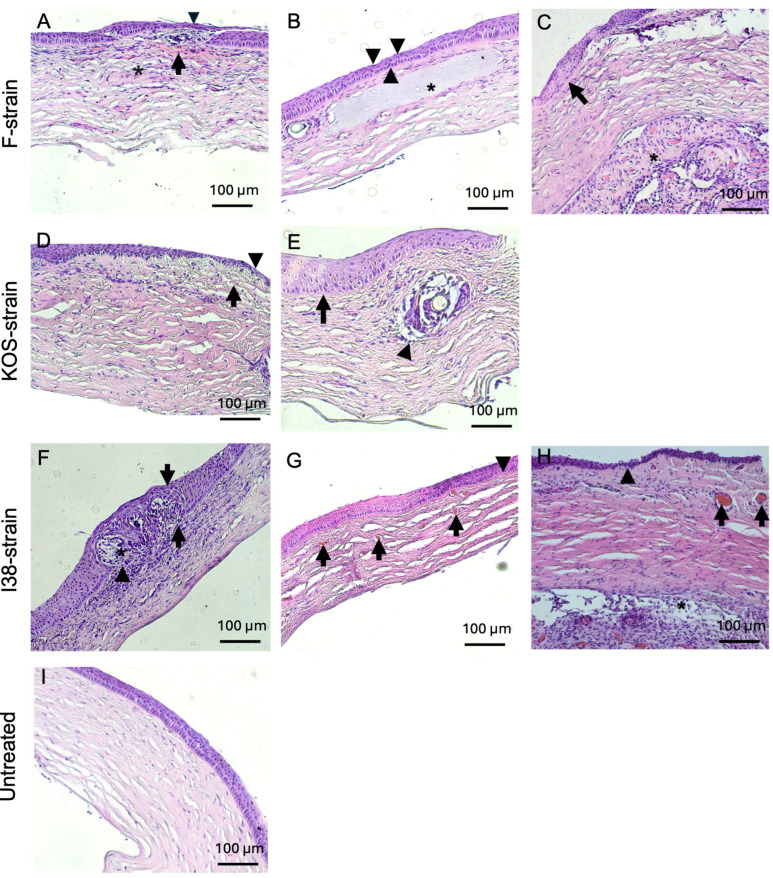
Hematoxylin and eosin-stained corneas from HSV-1-infected guinea pig corneas after CLP-PEG implantation at 3 months post-operation, compared with untreated control corneas. Representative corneas from F-strain-infected animals show a mild, diffuse, mixed inflammatory infiltrate (asterisk), hemorrhage (arrow), epithelial thinning (arrowhead), and mild to moderate stromal disorganization (**A**). Another representative cornea from an F-strain-infected guinea pig displays focal epithelial thinning (arrowheads) and moderate stromal disorganization (asterisk highlights the CLP-PEG implant within the corneal stroma) (**B**). Moderate epithelial hyperplasia (arrow) and retrocorneal fibrovascular proliferation and neovascularization (asterisk) were also observed in the F-strain-infected corneas (**C**). Corneas infected with the KOS strain showed mild stromal disorganization, neovascularization (arrow), and focal epithelial thinning (arrowhead) (**D**), as well as marked epithelial hyperplasia (arrow) and a mild mononuclear inflammatory infiltrate surrounding the area of the implant (arrowhead) (**E**). Corneas from guinea pigs infected with the i38 field strain presented a severe, mixed, diffuse, inflammatory infiltrate within the corneal stroma (arrowhead) with severe epithelial hyperplasia (arrows) and invasion of the epithelium (asterisk) and (**F**). Neovascularization (arrows) and moderate stromal disorganization was also observed (**G**). Another representative section of the i38 field strain group displayed epithelial thinning (arrowheads), neovascularization (arrows), and retrocorneal fibrovascular proliferation (asterisk) (**H**). Representative section of an untreated control cornea (**I**). Scale bars, 100 µm.

Histopathological examinations of H&E-stained rabbit corneal sections are presented in Figure 4 and Figure 5 and Table 4. Corneas from animals that died due to encephalitis were unavailable for the histopathological study. Two out of two rabbits infected with F-strain HSV-1 showed moderate to severe corneal inflammation characterized by infiltration of the corneal stroma by immune cells (Figure 4A,B), together with invasion and marked ulceration of the corneal epithelium. This was not observed in any of the rabbits infected with the KOS strain. KOS-infected rabbit corneas (Figure 4C,D) showed a tendency toward a more normal morphology, with a small number of blood vessels and some focal epithelial thinning.

Inflammatory infiltration was commonly observed in guinea pigs infected with all three HSV-1 strains used in this study (Figure 5A,D–F,H), with one animal infected with the i38 strain displaying a mixed inflammatory infiltrate at the level of the corneal epithelium (Figure 5F). The generation of new blood vessels within the corneal stroma was observed in 50% of the rabbits infected with the F strain and in 50% of the guinea pigs infected with the KOS and i38 strains (Figure 5D,G,H). Mild to moderate stromal disorganization and epithelial thinning were observed in corneas from both rabbits and guinea pigs infected with each of the virus strains (Figure 4 and Figure 5). Epithelial hyperplasia was also observed in rabbits infected with the KOS strain (Figure 4 D) and in guinea pigs infected with all three HSV-1 strains (Figure 5C,E,F). Two guinea pigs, one infected with the F strain and the other one infected with the i38 strain, also presented fibrovascular proliferation, together with neovascularization at the level of Descemet’s membrane (Figure 5C,H).

The untreated corneas of rabbits and guinea pigs from all experimental group showed normal histology by comparison (Figure 4E and Figure 5I).

## 4. Discussion

Collagen-analog-based CLP-PEG corneal implants were able to withstand the adverse conditions within HSV-1-infected rabbit and guinea pig corneas. They integrated seamlessly into the infected host corneas and promoted regeneration in 14 out of 14 animals. However, the active disease also left its evidence on the regenerated tissue to differing degrees in rabbits compared to guinea pigs. While mice have been used to study HSV-1 in various publications, the very small size of their corneas does not lend itself well to anterior lamellar keratoplasty for grafting corneal implants. However, rabbit and guinea pig corneas are large enough for such studies, and both have been used previously to study HSV-1 keratitis [30]. It has been proposed that HSK in guinea pigs more closely recapitulates human HSK than rodent and rabbit models [31,32].

When rabbit and guinea pig corneas were infected with HSV-1, a large proportion of the animals developed HSV-1 encephalitis (HSE), as previously reported [33,34,35]. HSE is a neurological complication that affects 2 to 4 people per 1,000,000 each year [35]. If left untreated, 70% of HSE patients die, while 20–30% mortality occurs even with treatment [36]. Many of the rabbits developed irreversible HSE, which limited their use in the corneal transplantation study. Prior research has shown that guinea pigs with an initial viral load similar to that used in our study demonstrated comparable ocular pathology, including corneal opacification and necrotizing keratitis [32]. In our hands, 58% of the rabbits and 16% of the guinea pigs developed HSE and were excluded from the study. The i38 clinical HSV-1 strain killed all four inoculated rabbits, while in guinea pigs, 50% (two out of four) survived. While these results suggested that rabbits are more sensitive to HSV-1 encephalitis than guinea pigs, the differences in proportions were statistically insignificant, possibly due to the small sample sizes of the animals. Nevertheless, the guinea pigs were able to develop corneal HSK after infection with the clinically isolated i38 virus, while all the rabbits died, lending support to the contention that guinea pigs might be a more appropriate model for human HSK than rodents or rabbits.

Analysis of the interaction between virus strain effects and species of animals showed no significant differences with respect to the development of encephalitis and mortality. However, detailed histopathological examination showed that F-strain-infected rabbit eyes showed greater abnormalities in their corneal epitheliums and more neovascularization compared to KOS-infected corneas, again suggesting the effect of viral strain differences at the microscopical level. A histopathological evaluation of guinea pig corneas showed very similar vascular abnormalities regardless of virus strain.

All initially cell-free corneal implants became populated with host epithelial and stromal cells, showing that the CLP-PEG hydrogels had stimulated in situ tissue regeneration, although the vascular lesions present showed active infection of the regenerated tissue. Nerve ingrowth was observed in F-infected guinea pig corneas, but not in those infected with the KOS or i38 strains. Angiogenesis has often been associated with nerve growth. In fact, VEGF-A, which is associated with angiogenesis, has been shown to promote corneal nerve regeneration [37].

In this study, while CLP-PEG was able to integrate within the infected corneas to promote regeneration, the regenerative process was hindered by the presence of the virus and inflammation. The resulting neocorneas showed re-establishment of the basic epithelial and stromal structure, albeit with disorganization that is seen in cell regeneration in chronically infected tissues [38].

Biomaterial-based systems incorporating antiviral peptides or drugs have shown superior outcomes in blocking viral activity while simultaneously promoting tissue regeneration [19,39]. For example, Cornea-in-a-Syringe (CIS)—an injectable hydrogel containing silica nanoparticles that release a modified cationic, anti-HSV-1 host defense peptide, GF19—is able to suppress inflammation and block peptide retention within rabbit corneas [39]. Incorporating antiviral agents into CLP-PEG implants or other biomaterials-based implants could, in the future, be a means to suppress viral reactivation and recurrence. More pivotal studies that possibly include delivery systems are, therefore, merited.

## 5. Conclusions

Despite the presence of an active virus, the implanted cell-free CLP-PEG hydrogels were sufficiently robust to remain stable within HSV-1-infected corneas and promoted the regeneration of corneal tissue. There was no statistical difference in rabbit vs. guinea pig survival in response to HSV-1 infection, but it was noted that no rabbits survived infection with a human clinically derived HSV-1. The guinea pig corneal HSV-1 infection model appears to be a more robust one. However, larger numbers of animals will need to be evaluated.

## Figures and Tables

**Table 1:** Different HSV-1 virus strains tested.

HSV-1 Strain	Origin	Source
F	Isolated from facial vesicle of a human patient.	ATCC #VR-733
KOS	Isolated from the lip lesion of a patient with a cold sore and deposited by the University of Pennsylvania.	ATCC# VR-1493
i38	Clinical strain from the eye of an HSK patient.	Univ. of Ottawa Eye Inst., Ottawa, Canada

**Table 2:** Physical and mechanical properties of CLP-PEG hydrogels, in comparison to the human cornea.

	CLP-PEG	Human Cornea
Water Content (%)	87.5 ± 3.87	78.0 ± 3.0 [24]
Refractive Index	1.343 ± 0.0015	1.373–1.380 [25]
White Light Transmission (%)	91.44 ± 4.78	87.1 ± 2.0 [26]
Backscatter (%)	0.65 ± 0.04 (%)	<3 [27]
Residual Mass (%)	15.82 ± 2.14 after 2 weeks	0 by 26 days [28]
DSC Tm (°C)	163	96
DSC Ttd (°C)	286	208
Storage Modulus G’(kPa)	31.13 ± 2.41	N/A
Linear Viscoelastic Region-LVR (%)	53 ± 7.81	N/A
Tensile Strength (kPa)	24.12 ± 3.38	3810 ± 400 [25]
Young’s Modulus (kPa)	41.14 ± 6.54	3000–13,000 [29]
Elongation at Break (%)	42.97 ± 2.12	N/A

N/A, not available.

**Table 3:** Characteristics of regenerated right neocorneas (OD) at three months pre- and post-operation compared to the uninfected, unoperated contralateral left corneas (OS).

Species (Number of Samples Examined)	Virus Strain	Eye	Haze Score (0–6) Pre-Op	Haze Score (0–6) Post-Op	Neovascularization Score (0–3) Pre-Op	Neovascularization Score (0–3) Post-Op	Pachymetry (µm) Post-Op	IOP (mmHg) Post-Op	Observations
Rabbit (*n* = 2)	F	OD	1 ± 0	2 ± 0	0.5 ± 0.7	2 ± 0	472.8 ± 48.9	3 ± 0	Distinct haze and vessels
		OS	0 ± 0	0 ± 0	0 ± 0	0 ± 0	427.0 ± 6.7	4 ± 0	
Rabbit (*n* = 3)	KOS	OD	0.33 ± 0.57	0.7 ± 0.6	0 ± 0	0.7 ± 0.6	350.4 ± 98.9	7 ± 2	Fine vessels and mild haze
		OS	0 ± 0	0 ± 0	0 ± 0	0 ± 0	396.1 ± 35.9	7.2 ± 0.3	
Guinea pig (*n* = 3)	F	OD	0.33 ± 0.57	0.7 ± 0.6	0.66 ± 0.6	1.7 ± 1.15	251.7 ± 27.7	6 ± 0	Very fine vessels over corneal surface and mild haze
		OS	0 ± 0	0 ± 0	0 ± 0	0 ± 0	224.5 ± 17.1	5.7 ± 1.1	
Guinea pig (*n* = 4)	KOS	OD	0 ± 0	0.25 ± 0.3	0 ± 0	2 ± 0	276.3 ± 84.3	5.5 ± 1.3	Mild haze, fine vessels over surface
		OS	0 ± 0	0 ± 0	0 ± 0	0 ± 0	227.4 ± 25.6	5 ± 0.8	
Guinea pig (*n* = 2)	i38	OD	0.5 ± 0.7	0 ± 0	0 ± 0	2 ± 0	280.5 ± 10.4	4.5 ± 0.7	Fine vessels over surface
		OS	0 ± 0	0 ± 0	0 ± 0	0 ± 0	242.2 ± 25.2	6 ± 1.4	

**Table 4:** Summary of histopathological results for HSV-1-infected corneas after CLP-PEG implantation at 3 months post-operation. Results are expressed as the percentage and number of sections with the corresponding lesion out of the total sections examined for each experimental group. Inflammatory infiltrate, stromal disorganization, and epithelial hyperplasia are characterized as 0 (absent), 1 (mild), 2 (moderate), and 3 (severe).

Evaluation Criteria	Rabbits			Guinea Pigs		
		F (*n* = 2)	KOS (*n* = 3)	F (*n* = 2)	KOS (*n* = 2)	i38 (*n* = 4)
Inflammatory infiltrate	0	-	100% [3/3]	50% [1/2]	-	-
	1	-	-	50% [1/2]	100% [2/2]	75% [3/4]
	2	50% [1/2]	-	-	-	-
	3	50% [1/2]	-	-	-	25% [1/4]
Neovascularization	No	50% [1/2]	100% [3/3]	100% [2/2]	50% [1/2]	50% [2/4]
	Yes	50% [1/2]	-	-	50% [1/2]	50% [2/4]
Stromal disorganization	0	50% [1/2]	33.33% [1/3]	-	50% [1/2]	-
	1	50% [1/2]	66.67% [2/3]	-	50% [1/2]	75% [3/4]
	2	-	-	100% [2/2]	-	25% [1/4]
	3	-	-	-	-	-
Epithelial thinning	No	-	-	50% [1/2]	-	50% [2/4]
	Yes	100% [2/2] *	100% [3/3]	50% [1/2]	100% [2/2]	50% [2/4]
Epithelial hyperplasia	0	100% [2/2]	33.33% [1/3]	50% [1/2]	50% [1/2]	-
	1	-	33.33% [1/3]	-	-	25% [1/4]
	2	-	33.33% [1/3]	50% [1/2]	-	25% [1/4]
	3	-	-	-	50% [1/2]	50% [2/4]
Other findings		100% [2/2] *	33.33% [1/3] ^†^	50% [1/2] ^‡^	50% [1/2] ^§^	75% [3/4] ^||^

* Both animals presented focal to extensive ulceration of the epithelium. ^†^ One of the animals presented multifocal hemorrhages within the corneal stroma. ^‡^ One of the animals presented with edema in the corneal stroma. ^§^ One of the animals presented retrocorneal fibrovascular proliferation with neovascularization. ^||^ One of the animals had multifocal hemorrhages within the corneal stroma, another one had retrocorneal fibrovascular proliferation with neovascularization, and a third had evidence for an inflammatory infiltrate at the level of the corneal epithelium.

## Data Availability

The original contributions presented in the study are included in the article, further inquiries can be directed to the corresponding authors.

## Abbreviations

The following abbreviations are used in this manuscript:

SARS-CoV-2	Severe acute respiratory syndrome coronavirus 2
HSV-1	Herpes simplex virus type 1
HSK	Herpes simplex keratitis
CLP	Collagen-like peptide
PEG	Poly(ethylene glycol)
MES	2-(N-morpholino)ethanesulfonic acid
EDC	1-ethyl-3-(3-dimethylaminopropyl) carbodiimide hydrochloride
NHS	N-hydroxysuccinimide
PBS	Phosphate-buffered saline
FTIR	Fourier-Transform Infrared Spectroscopy
ATR	Attenuated total reflectance
NMR	Nuclear magnetic resonance
FEG-SEM	Field emission gun scanning electron microscopy
Tris-HCl	Tris(hydroxymethyl)aminomethane hydrochloride
DSC	Differential scanning calorimetry
ALK	Anterior lamellar keratoplasty
IVCM	In vivo confocal microscopy
H&E	Hematoxylin and eosin
LVR	Linear viscoelastic region
HSE	Herpes simplex encephalitis
CIS	Cornea-in-a-Syringe
SiNP-GF19	Silica nanoparticles with cationic peptide GF19
